# Egg Nutriomics: Bridging Comprehensive Profiling and Precision Modulation of Bioactive Nutrient Factors in Eggs

**DOI:** 10.3390/foods15081330

**Published:** 2026-04-11

**Authors:** Hao Ding, Ziyi Wang, Jieyu Han, Yuehong Pang, Fei Liu, Xiaofang Shen

**Affiliations:** 1 School of Food Science and Technology, Jiangnan University, Wuxi 214122, China; 6230112134@stu.jiangnan.edu.cn (H.D.); wangziyi96723@163.com (Z.W.); hanjieyu1997@163.com (J.H.); yhpang@jiangnan.edu.cn (Y.P.); 2 Department of Modern Services, Yancheng Ocean Vocational School, Yancheng 224100, China; 3 Food Safety Detection Key Laboratory of Sichuan Province, Chengdu 610041, China

**Keywords:** egg nutriomics, visualization tools, multidimensional nutritional profiling, modulation of nutrient-enriched egg production

## Abstract

While global nutrient insufficiency remains a critical health challenge, eggs have emerged as a potential solution due to their profile as an accessible and nutrient-dense food source. To quantitatively assess this potential for mitigating nutrient insufficiencies and guide the production of nutrient-enriched eggs, the study proposes the concept of egg nutriomics, establishing a comprehensive evaluation system with 35 indicators across seven nutritional dimensions (fatty acids, amino acids, vitamins, trace elements, pigments, antioxidant capacity, and dietary restriction factors). Methodologically, the system normalizes raw analytical data into standardized scores (0–100) using indicator-specific functional models, with weights rationally allocated based on the essentiality of the nutrients. These quantitative metrics are subsequently translated into intuitive results using visualization tools such as heatmaps and radar charts. This study applied this system to evaluate six commercial egg varieties (pasteurized, lutein-enriched, ω-3 enriched, animal welfare, low-cholesterol, and conventional cage eggs), profiling multidimensional nutrition that allows for the intuitive visualization of performance scores across distinct dimensions. These profiles extend beyond comprehensive evaluation by revealing specific quantitative advantages—such as ω-3 enriched eggs scoring 79 in the fatty acid dimension compared to 49 for conventional eggs—thus providing a reference to guide precision modulation as illustrated by a dietary ω-3 enrichment case study involving 200 laying hens. Building upon this foundation, the strategy empowers a shift from the sole pursuit of high yields to precision nutritional modulation. This multi-dimensional strategy bridges nutritional analysis with production control, facilitating the development of nutrient-dense eggs as a potential application to mitigate human malnutrition.

## 1. Introduction

Although global food production has kept pace with population growth, more than 820 million people have insufficient food and many more consume low-quality diets that cause micronutrient deficiencies [1,2]. The burdens of malnutrition represent a critical challenge to human health, underscoring the importance of nutrient-dense food sources [3]. Eggs are widely consumed and nutrient-dense, providing a broad range of essential nutrients that support human health [4,5]. In addition to high-quality protein and lipids, eggs contain fatty acids, vitamins, minerals, carotenoids, and cholesterol, and the concentrations of these components can be modulated by the hen’s diet [6,7,8]. The egg market now offers a growing range of differentiated egg types aimed at addressing malnutrition, including products designed for specific nutritional goals such as ω-3 fatty acid-enriched eggs, lutein-rich eggs, and reduced-cholesterol eggs [9,10].

In the realm of addressing nutrient deficiencies, nutrient-enriched eggs emerge as a promising strategy [7]. However, their advent also poses significant challenges to the current quality assessment and regulatory frameworks. Current official egg grading relies largely on conventional quality metrics that describe physical quality but do not distinguish nutritional differences among nutrient-enriched egg products on the market. While the European Nutri-Score label attempts to evaluate food quality by classifying items from A to E based on a balance of positive and dietary restriction components, its assessment relies predominantly on energy and macronutrients [11]. Consequently, it overlooks micronutrients and bioactive compounds. More importantly, as a macroscopic model designed primarily for processed foods, Nutri-Score lacks the methodological sensitivity to capture the complex nutritional matrix inherent to whole foods. This represents a significant methodological gap, as such general-purpose labels cannot resolve the fine-scale nutritional heterogeneity among different egg types. In light of these limitations, there is an urgent imperative for the development of a comprehensive nutritional profiling system tailored specifically to eggs. Such a system should be capable of accurately quantifying the disparities in micronutrient content among different egg products. It would guide the transition from the traditional focus on yield optimization to a more sophisticated approach centered on precision nutritional modulation, thereby aligning with contemporary demands for improved nutritional outcomes.

Omics analyses, such as proteomics [12], metabolomics [13], lipidomics [14], and nutriomics [15,16], have been widely applied for their capacity to capture comprehensive compositional features of biological samples [17]. The nutriomics approach has been used to evaluate the nutritional value of different foods to address the limitations of single-indicator assessment [18]. Inspired by this, the study extends this approach to nutrient-enriched eggs to support nutritional profiling and production modulation.

This study proposes the concept of egg nutriomics—operationally defined as the holistic integration of comprehensive nutritional data (including micronutrients and bioactive compounds) to transcend traditional single-indicator assessments—establishing a multi-dimensional nutritional profiling system based on this concept. This comprehensive system encompasses both a scoring algorithm and weighting model. The scoring functions within this system are designed to align the average nutrient content in eggs with human nutritional needs. Concurrently, weights are allocated according to the relative significance of each individual nutrient. Through these integrated mechanisms, measured data can be transformed into readily interpretable results. The robustness of this developed system was validated through the characterization of the nutritional profiles exhibited by six distinct commercial functional egg varieties. Following this initial validation, the system was employed in a targeted trial focused on the enrichment of ω-3 fatty acids. The primary objective was to guide precision production modulation, thereby optimizing the nutritional quality of eggs. This strategy offers a framework that bridges agricultural production with human health demand.

## 2. Materials and Methods

### 2.1. Egg Samples

Six different groups of eggs were used in the experiment: pasteurized eggs (PE), lutein-enriched eggs (LE), ω-3 fatty acid-enriched eggs (OE), animal welfare eggs (AE), low-cholesterol eggs (LCE), and conventional cage eggs (CCE). These six groups were selected from different commercial brands. All samples were purchased simultaneously at a local large supermarket (Wuxi, China), ensuring they were produced within the same month, with 20 eggs collected per group. The eggs were immediately stored at 4 °C, and all nutritional profiling assays were completed within 10 days.

### 2.2. Measurement of Conventional Quality Indicators

The whole egg weight was measured using a precision analytical balance (Mettler Toledo ME204, Mettler, Greifensee, Switzerland, accuracy 0.001 g). The longitudinal and transverse diameters were then measured using a vernier caliper [19]. Eggshell color was measured using an eggshell color tester (ST-17, Shandong Shengtai Instrument Co., Ltd., Jinan, China), and eggshell strength was determined using an eggshell strength tester (BLD-DK09, Dongguan Bolaide Instrument Equipment Co., Ltd., Dongguan, China). Eggs were then broken onto a glass plate, and yolk color was scored using the Roche Color Fan. Albumen height was measured with an albumen height tester (XN-C2, Beijing Xinnuochen Instrument and Meter Co., Ltd., Beijing, China, accuracy 0.01 mm), and the Haugh unit was calculated accordingly as described by Haugh [20].

### 2.3. Determination of Amino Acids, Fatty Acids, Vitamins, and Pigments

Amino acid content was measured using the OPA pre-column derivatization reversed-phase high-performance liquid chromatography with ultraviolet detection (RP-HPLC-UV) and quantified using the external standard method. Briefly, 100 mg of homogenized sample was accurately weighed into a hydrolysis tube. After adding 8 mL of 6 mol/L hydrochloric acid solution, the tube was purged with nitrogen, sealed, and hydrolyzed at 120 °C for 24 h. After hydrolysis, the sample was cooled to room temperature, neutralized to approximately pH 7.0 using 10 mol/L sodium hydroxide solution, transferred to a 25 mL volumetric flask, and made up to volume with distilled water. An appropriate amount of the solution was filtered through double-layer filter paper, and the filtrate was centrifuged at 15,000 rpm for 30 min. Finally, 400 μL of the supernatant was placed in a vial for chromatographic analysis. The content of 37 fatty acids was determined using a gas chromatograph (Shimadzu, GC-2030, Kyoto, Japan) according to the first method described in GB 5009.168 [21]. The contents of Vitamin A, Vitamin B1, and Vitamin B2 were determined according to Chinese National Standards GB 5009.82 [22], GB 5009.84 [23], and GB 5009.85 [24], respectively. The contents of lutein and zeaxanthin in eggs were measured using the method described in Chinese Agricultural Industry Standard NY/T 3948-2021 [25]. These vitamins and color-related substances were quantified using a Waters e2695 HPLC system, with all results consistently expressed as mg/100 g.

### 2.4. Determination of Trace Elements, Antioxidant Capacity, and Cholesterol

The contents of Ca, Fe, Zn, Se, Cu, and Mn in eggs were determined by Inductively Coupled Plasma-Mass Spectrometry (ICP-MS, PerkinElmer, NexION 350D, Waltham, MA, USA) following microwave-assisted acid digestion according to the method described in Chinese National Standard GB 5009.268 [26]. For the DPPH radical scavenging assay, 5.00 g of the egg sample was accurately weighed and made up to 10 mL with absolute ethanol. The mixture was then centrifuged at 3500 r/min for 10 min and the supernatant was collected. The DPPH radical scavenging rate was determined by mixing the supernatant with a 50 μg/mL DPPH solution, followed by a 30 min incubation in the dark, with results expressed as a percentage (%) according to Chinese National Standard GB/T 39100-2020 [27]. Cholesterol content in whole eggs was measured according to the method described in Chinese National Standard GB/T 37077 [28] with results consistently expressed in mg/100 g. Both the DPPH radical scavenging and cholesterol assays were performed using a UV-Vis spectrophotometer (Shimadzu, UV-3600PLUS, Kyoto, Japan).

### 2.5. Procurement and Nutritional Analysis of Commercial Nutrient-Enriched Eggs

To validate the utility of the egg nutriomics framework in guiding the precision production of nutrient-enriched eggs, functional egg samples were directly purchased as commercial agricultural products from a commercial poultry company in Zibo, Shandong Province, China. These eggs were produced by 26-week-old Hy-Line Brown hens fed diets incorporated with graduated levels of chia seeds (0, 3.0%, 6.0%, 9.0%, 12.0%, 15.0%, 18.0%, or 21.0%). All purchased samples were immediately maintained at 4 °C to preserve nutritional integrity prior to nutritional profiling. The fatty acid composition was determined in accordance with the national standard method GB 5009.168.

### 2.6. Statistical Analysis

All experiments were conducted with three independent biological replicates to ensure data reliability and reproducibility. Prior to statistical evaluation, the assumptions of normality and homogeneity of variance were verified using the Shapiro–Wilk test and Levene’s test, respectively. Statistical analysis was performed using analysis of variance (ANOVA) by SPSS Statistics 20.0 software to assess significant differences between groups (*p* < 0.05), followed by Duncan’s test as a post hoc analysis. Graphing and partial data processing were completed using Origin 2021 software.

## 3. Results and Discussion

### 3.1. Standardized Criteria for Egg Quality Assessment

Standardized egg grading predominantly employs macroscopic physical metrics—including egg weight, shell integrity, and Haugh units—to assess quality and ensure market uniformity. This study assessed physical quality metrics in six commercial egg varieties (Table S3), all of which conformed to the Special Grade criteria under China National Standard GB/T 39438-2020 [29] (Table S4). However, this uniform classification reveals a critical limitation: current grading systems, while sufficient for ensuring safety and freshness, lack the discriminatory power to distinguish nutritional differences among eggs. Consequently, dependence on these conventional metrics provides insufficient guidance for consumers seeking to select eggs according to specific nutritional requirements. Therefore, it is imperative to construct a system that explicitly visualizes nutritional value.

### 3.2. Egg Nutriomics: A Multi-Dimensional Framework for Nutritional Profiling

Matching dietary intake to human physiological requirements remains a major challenge in functional food research. Eggs represent a nutrient-dense matrix characterized by diverse bioactive components—including amino acids [6], fatty acids [30,31], vitamins [32], pigments [33], trace elements [34], antioxidants [35], and cholesterol [36]. However, existing frameworks lack a comprehensive system to evaluate how well egg composition aligns with individual nutritional requirements. To address this gap, the study introduces the concept of egg nutriomics and establish a multidimensional profiling system based on 35 nutritional indicators spanning seven critical dimensions: amino acids, fatty acids, vitamins, trace elements, pigments, antioxidant capacity, and dietary restriction factors. To convert complex physicochemical data into intuitive, standardized metrics, the mathematical models were developed for each nutritional indicator (Table 1).

An arctangent-based scoring function was employed for beneficial nutrients to capture the non-linear relationship between concentration and nutritional value. For cholesterol, an inverse function was utilized to appropriately penalize excessive concentrations. These algorithms were calibrated using average nutrient values from the USDA FoodData Central [37] and the China Food Composition Tables [38], enabling normalization of raw measurements into a unified score scaled from 0 to 100. Specifically, the scoring models were anchored such that a nutrient concentration equivalent to these established average values corresponds to a baseline score of 60, representing a median nutritional proficiency that aligns with standard market expectations. Dimension scores were then computed through a weighted allocation strategy, integrating individual indicator scores according to the relative physiological importance of each nutrient to human health. For amino acids, the allocation was strictly hierarchical based on their nutritional indispensability: the limiting amino acids (e.g., methionine and lysine) were assigned the highest weights, and essential amino acids were consistently weighted higher than non-essential amino acids. Within the fatty acid profile, critical ω-3 polyunsaturated fatty acids, namely DHA and ALA, were evaluated as distinct parameters. Given its profound functional benefits for cardiovascular and neurological health, DHA was allocated the highest proportion of the weight among fatty acids. For minerals and trace elements, selenium and zinc were prioritized due to their crucial roles in antioxidant defense and immune function (which are primary targets for egg biofortification), followed by iron and calcium to address common public health shortfalls, while copper and manganese received lower weights reflective of their roles as lower-demand enzymatic cofactors. All other nutritional indicators within the framework were assigned equal weights to establish a balanced baseline. Finally, to validate the system’s evaluative capacity, this study applied it to profile six commercial egg varieties across all seven nutritional dimensions.

### 3.3. Nutritional Content Quantification and Scoring Within Seven Dimensions

**Figure 1:**
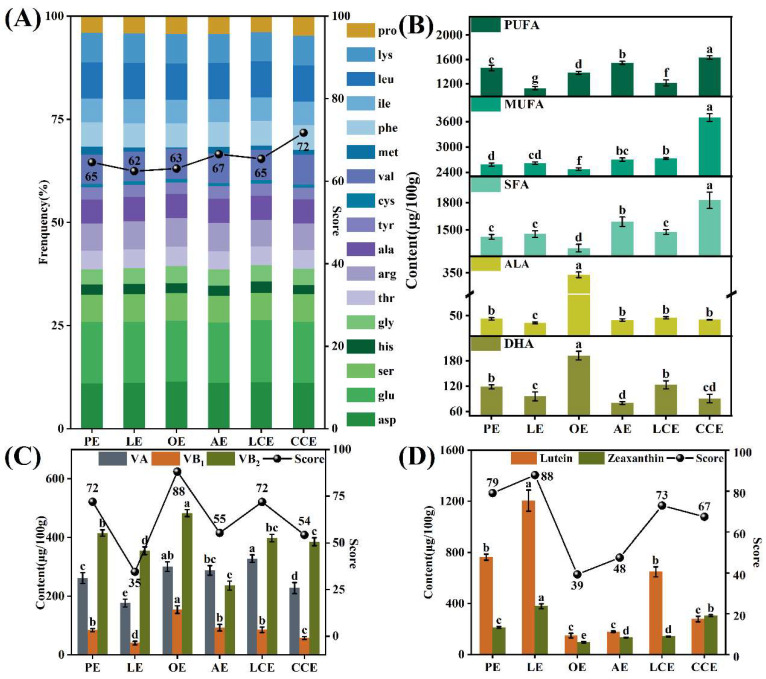
Comparison of nutritional components in six different types of eggs: (**A**) Amino acid composition and scoring; (**B**) Fatty acid components; (**C**) Vitamin content and scoring; (**D**) Carotenoid content and scoring. Letters indicate statistical differences (*p* < 0.05) between samples based on one-way ANOVA.

Leveraging the constructed evaluation system, the study quantified nutrient concentrations and computed standardized scores across seven nutritional dimensions for six commercial egg varieties, systematically profiling their nutritional heterogeneity. Eggs are a critical dietary source of protein, with an amino acid profile closely matching human physiological needs [6]. Consequently, eggs exhibit a biological value (BV) surpassing that of other high-quality animal proteins, including milk and beef [39]. However, this nutritional excellence is modulated by rearing conditions, such as dietary management and environmental factors. To address this variability, the study integrated amino acids as a core dimension into its evaluation system, systematically assessing protein quality across six egg varieties. As illustrated in Figure 1A, all tested egg varieties exceeded the 60-point threshold, which was defined using average amino acid composition data for standard eggs. Within the amino acid dimension, scores ranged from a minimum of 62 in the LE group to a maximum of 72 in the CCE group. This score distribution highlights inherent differences in protein profiles, validating the necessity of amino acids as an essential evaluative criterion.

Egg lipid composition can be modulated via dietary interventions to enrich specific functional fatty acids [30,31]. These lipids are classified into saturated fatty acids (SFAs), monounsaturated fatty acids (MUFAs) and polyunsaturated fatty acids (PUFAs), which collectively act as pivotal modulators of human health by influencing cardiovascular function, inflammatory pathways, and metabolic homeostasis. In this study, fatty acid concentrations across six egg varieties were quantified to assess their nutritional profiles (Figure 1B). Given the established link between excessive SFA intake and cardiovascular risk, an inverse scoring function was applied to this indicator. Under this framework, the highest SFA concentration was observed in CCE (1828.19 mg/100 g; score: 66), while the lowest occurred in OE (score: 89). MUFA levels were highest in CCE (3693.04 mg/100 g; score: 78), contrasting sharply with OE’s minimum value (score: 30). For total PUFA, CCE again ranked highest (1628.94 mg/100 g; score: 57), whereas LE scored lowest (1127.64 mg/100 g; score: 45). Within the egg nutriomics framework, α-linolenic acid (ALA) and docosahexaenoic acid (DHA) are prioritized as key functional indicators due to their roles in cardiovascular health and neurodevelopment. DHA concentration was highest in OE (192.64 mg/100 g; score: 85), alongside the highest ALA level (345.28 mg/100 g; score: 98). Conversely, AE exhibited the lowest DHA content (79.89 mg/100 g; score: 28), while LE had the minimum ALA (32.37 mg/100 g; score: 32). These findings highlight the discriminatory power of fatty acid profiling, confirming its critical role in multidimensional nutritional assessment.

Vitamins are essential for physiological function and metabolic regulation, and their concentrations in eggs can be substantially enhanced via hen dietary supplementation [30,32]. Given this enrichment capacity, key vitamins (A, B_1_, and B_2_) were quantified across six commercial egg varieties, with comparative profiles presented in Figure 1C. Vitamin A levels were highest in LCE (328.37 μg/100 g; score: 89) and OE (301.06 μg/100 g; score: 87), whereas LE exhibited the lowest concentration at 176.87 μg/100 g (score: 48). The highest concentration of vitamin B1 was observed in the OE group (154.78 μg/100 g; score: 91), while the lowest occurred in the LE group (40.63 μg/100 g; score: 22). Similarly, vitamin B2 levels were highest in OE (483.05 μg/100 g; score: 87), contrasting sharply with the minimum value in AE (236.51 μg/100 g; score: 20). Within the overall vitamin dimension, OE achieved the highest composite score (88), followed by LCE. Notably, despite its premium market positioning relative to CCE (score: 54), the LE variety received a substantially lower nutritional score (35), highlighting a disconnect between commercial valuation and the nutritional value of the vitamin dimension. This significant variation necessitates vitamin profiling to fully reflect the nutritional breadth across different varieties.

Yolk color is a primary sensory determinant of consumer preference and perceived quality [33], with visual assessments using the Roche Color Fan revealing a distinct hierarchical gradient in pigmentation intensity (Table S3). This visual profile arises from the deposition of fat-soluble bioactive compounds, such as lutein and zeaxanthin, which contribute to vision preservation, neurological function, and systemic antioxidant defense [40,41]. In this study, lutein and zeaxanthin concentrations were quantified across six commercial egg varieties, with results summarized in Figure 1D. For lutein, the highest concentration occurred in the LE group (1262.67 μg/100 g; score: 93), while the lowest was observed in OE (149.01 μg/100 g; score: 44). Similarly, zeaxanthin levels were highest in LE (379.65 μg/100 g; score: 76) and reached their minimum in OE (97.33 μg/100 g; score: 29). Notably, despite the LCE group exhibiting the most intense visual pigmentation (Table S3), its corresponding pigment dimension score was 73. This discrepancy is likely attributable to the influence of authorized pigment additives, which enhance chromatic intensity without contributing to functional carotenoid content. Together, these findings demonstrate that natural carotenoid profiling provides a more accurate metric for nutritional valuation than subjective visual grading.

**Figure 2:**
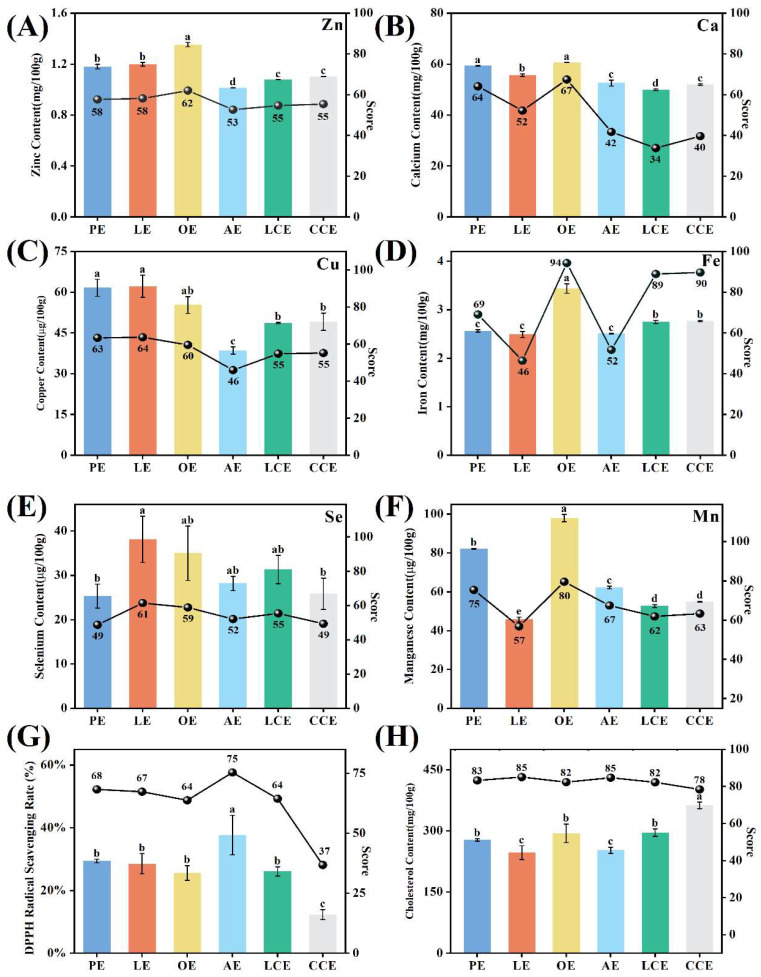
Content and scoring results of various indicators in six different types of eggs: Zn (**A**), Ca (**B**), Cu (**C**), Fe (**D**), Se (**E**), Mn (**F**), DPPH scavenging rate (**G**), and Cholesterol (**H**). Letters indicate statistical differences (*p* < 0.05) between samples based on one-way ANOVA.

Trace elements are essential for maintaining metabolic homeostasis and overall health, and eggs represent an important dietary source of these micronutrients [34]. Given that egg elemental composition is directly modulated by hen diet, this study profiled key trace elements (zinc, calcium, copper, iron, selenium, and manganese) across six commercial varieties to assess their nutritional densities (Figure 2A–F). Calcium concentrations were highest in the OE group (60.59 mg/100 g; score: 67) and were lowest in LCE (49.89 mg/100 g; score: 34). Iron levels were highest in OE (3.44 mg/100 g; score: 94) but lowest in LE (2.49 mg/100 g; score: 46). Zinc content reached its maximum in OE (1.35 mg/100 g; score: 62) and minimum in AE (1.01 mg/100 g; score: 53). Manganese abundance similarly was highest in OE (97.93 μg/100 g; score: 80) and reached its nadir in LE (45.79 μg/100 g; score: 57). Selenium concentrations were highest in LE (38.10 μg/100 g; score: 61) and lowest in PE (25.34 μg/100 g; score: 49), while copper levels were highest in LE (62.20 μg/100 g; score: 64) and were minimized in AE (38.54 μg/100 g; score: 46). The observed heterogeneities underscore the essential role of trace element analysis in achieving a truly comprehensive nutritional assessment.

The total antioxidant capacity of eggs reflects the synergistic action of diverse bioactive constituents that are associated with a reduced risk of coronary heart disease and stroke [34,35]. To evaluate this integrated antioxidant potential, DPPH radical scavenging capacity was quantified as a standardized in vitro metric [42] across six commercial varieties (Figure 2G). For the antioxidant capacity, the highest value was observed in the AE group (37.7%; score: 75), while the lowest occurred in the CCE group (12.3%; score: 37). The elevated antioxidant response in AE may be linked to its rearing system, aligning with prior evidence that free-range eggs contain higher levels of antioxidant-related compounds compared to those from cage systems [43]. The pronounced heterogeneity observed in these scores validates the antioxidant dimension as an essential metric within the system.

The high cholesterol content of eggs has long been a focal point in dietary guidelines and a primary consumer concern [36]. The cholesterol concentration in eggs is significantly modulated by hen age, rearing systems, and dietary interventions. Reflecting its critical implications for cardiovascular health, cholesterol was included as a dietary restriction metric, with concentrations for six commercial egg varieties presented in Figure 2H. To reflect its physiological impact, cholesterol was scored using an inverse function based on the USDA reference value for conventional eggs (411 mg/100 g), such that higher concentrations yielded lower nutritional scores. For the cholesterol indicator, the highest concentration occurred in the CCE group (362.70 mg/100 g; score: 78), while the lowest levels were observed in both LE and AE groups (score: 85). Notably, the LCE exhibited a cholesterol concentration of 295.73 mg/100 g (score: 82), surpassing several counterparts without low-cholesterol labeling. This discrepancy highlights a misalignment between commercial claims and objective measurements. This discrepancy may occur because commercial “low-cholesterol” claims are compared with other standards rather than standard caged eggs. Therefore, incorporating this dimension is essential to reveal discrepancies between commercial low-cholesterol claims and the actual cholesterol levels sought by consumers, thereby enabling an objective assessment of egg cholesterol content.

Applying the scoring models, measured nutrient data were transformed into standardized scores (0–100), revealing nutritional strengths and weaknesses independent of absolute concentrations. However, the interpretation of extensive numerical datasets remains insufficient to assist consumers in selecting products tailored to their nutritional needs or to guide the strategic production of functional eggs. Therefore, the implementation of heatmaps and radar charts is essential to distill these metrics into interpretable patterns, enabling consumers to intuitively discern the nutritional value of eggs across various dimensions. The pronounced variations observed across these metrics validate the egg nutriomics as a superior strategy for nutritional evaluation. Furthermore, the development of mathematically grounded scoring models forms the essential basis for comprehensive profiling, effectively translating complex analytical outcomes into intuitive data.

### 3.4. Multidimensional Nutritional Score Visualized by Heatmaps and Radar Charts

**Figure 3:**
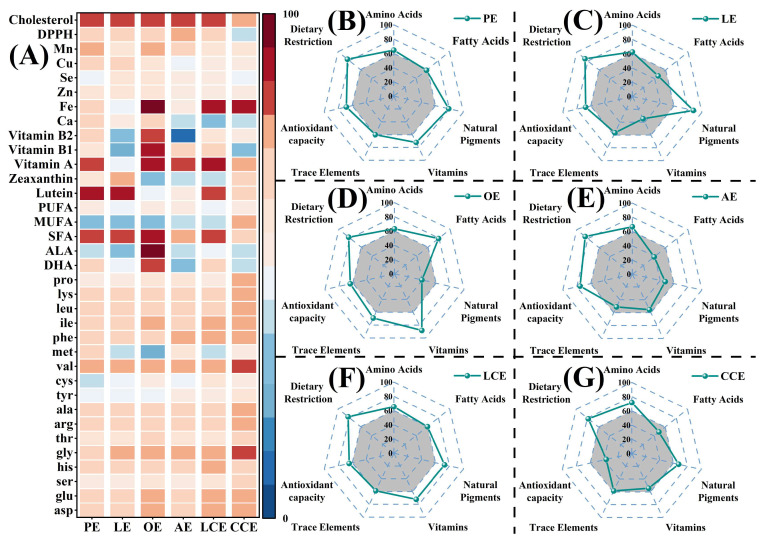
Visualization of multi-dimensional nutritional characteristics of six egg types: (**A**) Heatmap of 35 nutritional indicators; (**B**–**G**) Radar charts of the six egg types across seven nutritional dimensions.

Serving as the core computational algorithm within the egg nutriomics framework, the scoring model converts raw nutrient concentrations into standardized nutritional value scores (ranging from 0 to 100, where higher scores generally reflect better alignment with physiological needs). To visualize these multidimensional datasets effectively, heatmaps and radar charts were utilized to clearly delineate the distinct nutritional profiles of each variety (Figure 3). The heatmap summarizes performance across 35 nutritional indicators, where deeper red hues represent higher scores (approaching 100), and lighter hues represent lower scores, enabling rapid identification of relative strengths and weaknesses for each variety (Figure 3A). OE demonstrated elevated scores for ω-3 PUFA-related indicators and strong performance in vitamin metrics. LE was characterized by superior scores for lutein and zeaxanthin indicators, while AE excelled in antioxidant capacity. CCE exhibited lower antioxidant capacity scores but relatively higher amino acid scores. LCE achieved comparatively higher scores for vitamin A and iron. This heatmap approach facilitates indicator-level comparison, revealing relative differences among products and highlighting the unique nutritional signatures of each egg group.

To intuitively visualize the nutritional heterogeneity across the egg varieties, dimension-level total scores were calculated using weighted allocation and visualized via radar charts (Figure 3B–G). In these plots, the distance from the center along each axis corresponds to the aggregated score (0–100) for a given dimension, with a larger outward expansion indicating a superior overall nutritional profile in that category. These visualizations enabled direct comparison of relative strengths across seven key dimensions: amino acids, fatty acids, pigments, vitamins, trace elements, antioxidant capacity, and dietary restriction factors. OE exhibited high scores in the fatty acid (79) and vitamin (88) dimensions. LE achieved the highest pigment score (88) but the lowest vitamin score (35). AE attained the top antioxidant capacity score (75). PE and LCE displayed relatively balanced profiles across dimensions, whereas CCE recorded the highest amino acid score (72) yet the lowest antioxidant capacity score (37), highlighting distinct trade-offs between nutrient categories. By graphically comparing the distinct nutritional strengths and weaknesses of each variety, these visualizations provide an intuitive tool to guide consumers toward products aligned with their nutritional needs.

**Figure 4:**
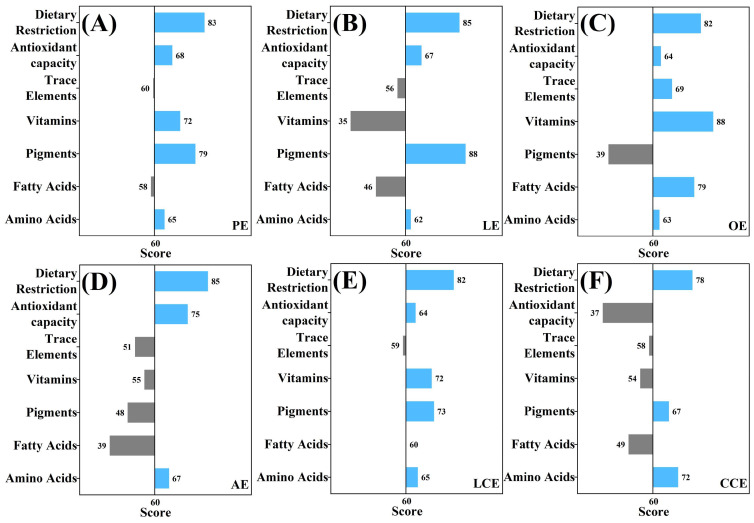
Multi-dimensional nutritional assessment scores of six commercial egg varieties relative to the average baseline: (**A**) PE; (**B**) LE; (**C**) OE; (**D**) AE; (**E**) LCE; (**F**) CCE.

Figure 4 illustrates the multidimensional nutritional evaluation of six nutrient-enriched egg varieties relative to a standardized benchmark. With 60 points established as the nutritional baseline, bars extending to the right signify levels exceeding the average for conventional eggs, indicating successful enrichment. Conversely, bars extending to the left denote dimensions that fall short of industry averages, highlighting specific nutritional deficiencies. The result reveals that all varieties consistently remain to the right of the baseline in both dietary restriction (cholesterol) and amino acid dimensions. However, none of the six commercial varieties achieved universal proficiency across all evaluated dimensions. Notably, PE and LCE demonstrated superior nutritional balance, characterized by an absence of significant nutritional deficits. Single-nutrient enriched eggs exhibit marked nutritional imbalances across nutritional dimensions. For instance, while the LE variety demonstrates exceptional performance in natural pigments, it reveals a significant negative divergence in its vitamin profile. Consequently, the production of nutrient-enriched eggs necessitates a shift from a focus on target nutrients to a comprehensive management of nutritional value. The application of egg nutriomics holds significant practical implications, enabling precise assessment of nutritional value during production and providing a theoretical basis for targeted interventions and the development of nutritionally enhanced, high-quality eggs.

This multi-dimensional system leverages the egg nutriomics to facilitate a holistic assessment of nutritional value. Demonstrating strong descriptive and discriminative capabilities across six commercial egg varieties, this multi-dimensional system integrates function-based scoring, weighted allocation, and visualization to holistically assess nutritional value. By intuitively presenting these metrics, the approach bridges consumer and producer needs: it enables personalized purchasing decisions aligned with nutritional requirements while guiding targeted feed optimization based on identified nutritional deficits and strengths.

### 3.5. Egg Nutriomics for Precision Production Exemplified by ω-3 PUFA Enrichment as a Case Study

**Figure 5:**
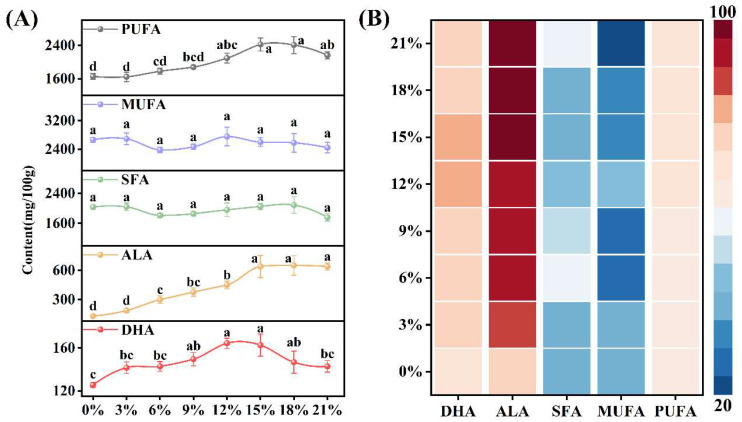
Effects of different dietary chia seed supplementation levels on fatty acid content (**A**) and nutritional scoring (**B**) in eggs. Letters indicate statistical differences (*p* < 0.05) between samples based on one-way ANOVA.

Egg nutriomics facilitates the precise modulation of production by monitoring changes in egg nutritional profiles and guide the reverse regulation of feed formulations. Chia seed oil serves as a potent source of ALA, with ALA comprising approximately 62% of the total fatty acid [44]. This unique lipid profile makes it an ideal dietary ingredient for validating the precise modulation of ω-3 PUFA-enriched eggs. In this study, the fatty acid nutritional profiles and dimension scores of procured commercial ω-3-enriched eggs were systematically monitored to evaluate the shifts occurring under different chia seed inclusion levels (Figure 5). SFA and MUFA levels remained unaffected by chia seed supplementation (*p* > 0.05), likely owing to the low concentrations of these fatty acids within the seeds. ALA content increased dose-dependently from 131.79 mg/100 g in the control group to 639.63 mg/100 g at 21% inclusion, with corresponding scores rising from 75.5 to 97. DHA concentrations were highest at 164.45 mg/100 g, exceeding the control value of 108.74 mg/100 g, at 12.0% inclusion and achieved a score of 81; higher inclusion levels did not further elevate DHA. At the optimal inclusion level of 12.0%, the ALA-related score reached 95. Further increases in chia seed supplementation elevated ALA content but induced only marginal score changes, differing by approximately two points. This result underscores the limitations of prioritizing maximal absolute concentrations. Unlike conventional methods focused solely on target analyte levels, this strategy identified 12.0% as the optimal supplementation threshold under the specific feeding conditions detailed in this study. Through integrated fatty acid profiling, the study thereby validates egg nutriomics as a tool for precision modulation of nutrient-enriched eggs. By enabling dynamic monitoring of nutritional variations, egg nutriomics bridges comprehensive profiling with targeted production interventions, offering actionable guidance for optimizing dietary formulations.

## 4. Conclusions

In conclusion, this study introduces the egg nutriomics concept, establishing a systematic evaluation framework encompassing 35 nutritional indicators across seven dimensions. By transforming complex analytical data into intuitive heatmaps and radar charts, the system enables dual functionality: guiding consumer choices toward personalized nutritional needs while facilitating precision production modulation. This work establishes a standardized paradigm for multi-dimensional nutritional assessment, providing a scalable framework for the comprehensive evaluation of nutrient-enriched eggs. While the current findings successfully highlight the system’s ability to discriminate among different products, future studies will be required to establish a true methodological validation of the proposed model. As the variety of nutrient-enriched eggs broadens, future evaluation systems will evolve to integrate a more comprehensive array of nutrients. By establishing a system to enhance nutrient density, this work offers a valuable tool to support the development of nutrient-dense foods and contribute to improved public health outcomes.

## Figures and Tables

**Table 1:** Dimensions, indicators, scoring functions, and weights of the egg nutriomics profiling system.

Nutritional Dimension	Indicator	Scoring Formula	Weight/%
Amino acid composition	Aspartic acid	y(x) = 30 × arctan(0.21 × (x − 11.5)) + 53	2
	Glutamic acid	y(x) = 30 × arctan(0.16 × (x − 14.8)) + 53	3
	Serine	y(x) = 30 × arctan(0.31 × (x − 8.4)) + 53	2
	Histidine	y(x) = 30 × arctan(0.86 × (x − 2.5)) + 53	6
	Glycine	y(x) = 30 × arctan(0.61 × (x − 3.7)) + 53	2
	Threonine	y(x) = 30 × arctan(0.41 × (x − 5.3)) + 53	7
	Arginine	y(x) = 30 × arctan(0.31 × (x − 7.1)) + 53	6
	Alanine	y(x) = 30 × arctan(0.36 × (x − 6.0)) + 53	3
	Tyrosine	y(x) = 30 × arctan(0.51 × (x − 4.6)) + 53	5
	Cysteine	y(x) = 30 × arctan(0.66 × (x − 3.5)) + 53	4
	Valine	y(x) = 30 × arctan(0.36 × (x − 6.7)) + 53	8
	Methionine	y(x) = 30 × arctan(0.61 × (x − 3.8)) + 53	12
	Phenylalanine	y(x) = 30 × arctan(0.41 × (x − 6.0)) + 53	7
	Isoleucine	y(x) = 30 × arctan(0.41 × (x − 5.6)) + 53	8
	Leucine	y(x) = 30 × arctan(0.23 × (x − 9.5)) + 53	10
	Lysine	y(x) = 30 × arctan(0.29 × (x − 7.5)) + 53	12
	Proline	y(x) = 30 × arctan(0.43 × (x − 5.0)) + 53	3
Fatty acids	Docosahexaenoic acid (DHA)	y = 51 × arctan(0.028 × (x − 14.6)) + 20	50
	Alpha-linolenic acid (ALA)	y = 48 × arctan(0.021 × (x − 30)) + 30	20
	Monounsaturated fatty acid (MUFA)	y = 35 × arctan(1.5 × (x − 2.5)) + 46	10
	Polyunsaturated fatty acid (PUFA)	y = 64 × arctan(0.76x)	10
	Saturated fatty acid (SFA)	y = −38 × arctan(1.8 × (x − 2)) + 55	10
Pigments	Lutein	y = 51 × arctan(0.0062 × (x − 67.4)) + 20	50
	Zeaxanthin	y = 51 × arctan(0.0062 × (x − 67.1)) + 20	50
Vitamins	Vitamin A	y = 32 × arctan(0.020 × (x − 180.9)) + 50	33
	Vitamin B_1_	y = 35 × arctan(0.062 × (x − 70)) + 48	33
	Vitamin B_2_	y = 32 × arctan(0.021 × (x − 380)) + 50	33
Trace elements	Ca	y = 32 × arctan(0.11 × (x − 55)) + 50	15
	Fe	y = 51 × arctan(3.95 × (x − 2.23)) + 20	19
	Zn	y = 51 × arctan(0.99 × (x − 0.26)) + 20	23
	Se	y = 64 × arctan(0.038x)	28
	Cu	y = 51 × arctan(0.025 × (x − 16.40)) + 20	9
	Mn	y = 51 × arctan(0.028 × (x − 14.64)) + 20	6
Dietary restriction factors	Cholesterol	y = 5,856,000/(x + 9787) − 500	100
Antioxidant capacity	DPPH radical scavenging rate	y = 51 × arctan(6.15 × (x − 0.067)) + 20	100

## Data Availability

The original contributions presented in this study are included in the article/supplementary material. Further inquiries can be directed to the corresponding authors.

## Supplementary Materials

The following supporting information can be downloaded at: https://www.mdpi.com/article/10.3390/foods15081330/s1, Table S1: Composition and nutrient levels of basal diets (air-dried basis, %); Table S2: Diet compositions and nutrient contents ^1^ (%); Table S3: Conventional quality indicators and retail prices of six commercial egg varieties; Table S4: Grading Requirements for Egg Quality; Table S5: Effect of dietary chia seed levels on production performance and egg traits for laying hens after 8 weeks of feeding ^1^.; Table S6. Fatty acids composition of experimental diets ^1^ (mg/g, as-is basis); Table S7: Effect of dietary chia seed levels on yolk, albumen, and eggshell content in eggs for laying hens after 8 weeks of feeding.

## Abbreviations

The following abbreviations are used in this manuscript:

SFAs	Saturated fatty acids
MUFAs	Monounsaturated fatty acids
PUFAs	Polyunsaturated fatty acids
ALA	α-linolenic acid
DHA	Docosahexaenoic acid
