# Supplementary material for: Egg Nutriomics: Bridging Comprehensive Profiling and Precision Modulation of Bioactive Nutrient Factors in Eggs
Source: Foods. 2026 Apr 11;15(8):1330. doi: 10.3390/foods15081330 (PMC13115482; doi:10.3390/foods15081330)
Supplement: Supplementary file 1 [file foods-15-01330-s001.zip › foods-4201173-supplementary.pdf]

Supporting Information for

Egg Nutriomics: Bridging Comprehensive Profiling and Precision Modulation of Bioactive Nutrient Factors in Eggs

Hao Ding[1], Ziyi Wang[1,2], Jieyu Han[1], Yuehong Pang[1], Fei Liu[1,*] and Xiaofang Shen[1,3,*]

[1]*School of Food Science and Technology, Jiangnan University, Wuxi 214122, PR China*

[2]*Department of Modern Services, Yancheng Ocean Vocational School, Yancheng 22400, PR China*

[3]*Food Safety Detection Key Laboratory of Sichuan Province, Chengdu, 610041, PR China*

**Corresponding Author:*

Fei Liu, E-mail: feiliu@jiangnan.edu.cn

Xiao-Fang Shen, E-mail: xfshen@jiangnan.edu.cn

SUPPORTING TABLES

At a commercial laying hen farm in Zibo, Shandong Province, China, a total of 200 26-week-old Hy-Line Brown laying hens were randomly allocated into 8 groups, with 5 replicates per group and 5 hens per replicate. The feeding trial lasted for 8 weeks. To mitigate environmental and positional effects, hens were randomly assigned to cage positions by number. Hens had ad libitum access to feed and water. The ambient temperature was maintained at 20 ± 2°C, and relative humidity was 50-60%. Ventilation was managed using a combination of natural and vertical negative pressure ventilation. The lighting schedule consisted of natural light supplemented with artificial light (16 h/day) at an intensity of 20 lux. Manure was removed twice daily, routine immunization and disinfection were performed weekly, feed was added regularly with diligent recording, and eggs were collected twice daily.

Table S1 Composition and nutrient levels of basal diets (air-dried basis, %).

Items	Content	Nutrients levels	
Corn	51.00	ME (MJ/kg)[2]	11.60
Soybean meal	31.10	Crude protein (%)[3]	16.82
Limestone	1.40	Calcium (%)[3]	4.24
$CaHPO_4$	8.70	Total phosphorus (%)[3]	0.85
Premix[1]	4.00	Methionine (%)[3]	0.46
Soybean Oil	3.80	Lysine (%)[3]	0.88
Total	100.00		

[1]Provided the following per kilogram of diet: vitamin A, 150000-200000 IU; vitamin D, 50000-100000 IU; vitamin E, 300 mg; vitamin K, 70 mg; vitamin B_1,4.5 mg; vitamin B_2, 140 mg; vitamin B_6, 80 mg; vitamin B_{12}, 0.4

mg; nicotinamide, 550 mg; calcium pantothenate, 200 mg; folic acid, 11 mg; biotin, 2 mg; choline chloride, 3 g; Cu, 80 mg; Fe, 500 mg; Zn, 500 mg; Mn, 600 mg; I, 10000 mg; Se, 1000 mg; Ca, 5%; NaCl, 6%; moisture content, 10%; Methionine, 2.5%.

[2]Values were calculated according to MEn of feedstuffs for poultry provided by NRC (1994) and NY/T 33-2004.

[3]The numbers were analyzed values.

Table S2 Diet compositions and nutrient contents[1] (%).

Items	Control	Chia seed supplemental levels (%)						
		3	6	9	12	15	18	21
ME(MJ/kg)	11.60	11.66	11.73	11.80	11.86	11.93	12.00	12.07
Lysine	0.88	0.89	0.90	0.91	0.93	0.94	0.95	0.96
Methionine	0.46	0.46	0.47	0.48	0.48	0.49	0.49	0.50
Methionine + cystine	0.54	0.57	0.60	0.62	0.65	0.68	0.71	0.73
Threonine	0.60	0.61	0.62	0.63	0.64	0.65	0.66	0.67
Tryptophan	0.23	0.24	0.25	0.26	0.27	0.27	0.28	0.29
Arginine	0.99	1.03	1.07	1.12	1.16	1.21	1.25	1.30
Isoleucine	0.62	0.62	0.63	0.64	0.64	0.65	0.66	0.66
Valine	0.72	0.73	0.74	0.75	0.76	0.77	0.78	0.79
Crude protein	16.82	16.93	17.04	17.15	17.26	17.37	17.48	17.59
Na	0.34	0.33	0.32	0.31	0.31	0.30	0.29	0.28
Ca	4.24	4.14	4.03	3.93	3.83	3.73	3.63	3.52
P	0.85	0.85	0.84	0.83	0.83	0.82	0.81	0.81

[1]The numbers were analyzed values.

Table S3 summarizes the conventional quality indices of the six egg types, showing only modest variation in egg weight across groups, while the shape index separated OE above 76 and CCE below 72, with the other groups falling within 72–76, which is considered suitable for commercial handling and circulation. Eggshell hardness exceeded 30 N in all groups, indicating comparable resistance to mechanical stress during transport and storage. Yolk color was scored using the Roche Color Fan, with OE and LCE showing higher scores of 13.7 and 14.7, PE, LE, and AE scoring 12.3, 12.3, and 12.0, and CCE having the lowest score of 9.70. Eggshell color exhibited clearer variation across groups when quantified using the CIELAB system. LCE exhibited a lower L* value than the other groups, indicating a darker shell appearance, while the a* and b* values suggested broadly similar reddish-yellow hues across groups except for CCE. Conventional indicators describe external quality and basic circulation suitability, but they provide limited resolution for differentiating nutritional composition across egg types.

Table S3 Conventional quality indicators and retail prices of six commercial egg varieties.

Item		PE	LE	OE	AE	LCE	CCE
Egg Shape Index		73.91±3.58bc	73.81±1.73bc	79.37±1.10a	76.92±0.74ab	73.08±2.22cd	70.13±2.65d
Egg Weight(g)		56.12±5.39a	56.53±1.22a	60.30±0.96a	63.45±1.35a	58.71±0.78a	45.14±0.52b
Eggshell Strength(N)		43.67±4.69bc	53.23±5.40ab	55.36±4.68a	45.18±5.57bc	50.37±4.00abc	44.39±8.09bc
Yolk Color		12.30±0.60c	12.30±0.60c	13.70±0.60b	12.00±0.00c	14.70±0.60a	9.70±0.60d
Haugh Unit		74.86±4.18c	81.86±0.99ab	82.88±0.58a	81.71±1.24ab	82.96±1.16a	77.89±0.87bc
Eggshell Color	a^*	4.70±0.43c	5.08±0.48c	3.41±0.20d	7.31±0.31b	8.96±0.18a	3.32±1.31d
	b^*	15.26±1.10cd	15.15±0.61d	16.36±0.46c	21.59±0.84a	19.59±0.29b	15.57±0.21cd

L^*	82.55±0.74bc	82.96±0.75ab	84.70±0.55a	78.67±0.99d	75.80±0.42e	81.06±2.04c
Price per egg	2.5 yuan	1.1 yuan	2.8 yuan	1.6 yuan	2.2 yuan	0.66 yuan

$^{a-d}$- different letters in the columns show that there is a significant difference between the values ($p < 0.05$).

Table S4. Grading Requirements for Egg Quality (GB/T 39438-2020)

Indicator		Special Grade	Grade I	Grade II
Appearance	Shell Quality	Possesses the inherent color of the variety; shell is intact without breakage; free from obvious spots, rough texture, or malformations		
	Shell Cleanliness	No visible stains on the shell surface		Visible stains allowed; single stain area ⩽ 4 mm^2, and total stained area ⩽ 8 mm^2

Internal Content	Yolk	Intact; no loose yolk.		
	Haugh Unit	> 72	> 60	> 55
	Albumen	Viscous and transparent; thick and thin albumen clearly distinguishable	Moderately viscous and transparent; thick and thin albumen clearly distinguishable	Moderately viscous and transparent
	Germinal Disc	No obvious development visible		
	Foreign Matter	Blood spots or meat spots with a diameter < 2 mm are permitted; no other foreign matter allowed.		

Table S5. Effect of dietary chia seed levels on production performance and egg traits for laying hens after 8 weeks of feeding.[1]

Items	Control	Chia seed supplemental levels (%)							SEM[2]	*P*-value
		3	6	9	12	15	18	21		
Egg production (%)	92.00±8.00	100.00±0.00	100.00±0.00	100.00±0.00	91.00±5.57	100.00±0.00	87.00±8.31	92.00±4.90	1.878	0.331
Average egg weight (g)	63.08±0.61^{abc}	61.78±0.84^{cd}	63.34±0.59^{abc}	63.02±0.81^{abc}	64.68±0.61^{a}	64.27±1.29^{ab}	61.96±1.09^{bcd}	60.66±1.00^{d}	0.369	0.008
Daily feed intake (g/bird)	128.88±7.47	134.04±4.86	136.72±4.32	127.41±6.14	138.47±5.87	127.20±12.63	131.07±5.05	132.92±6.75	1.487	0.835
Feed conversion rate	2.25±0.10^{ab}	2.17±0.08^{ab}	2.16±0.07^{ab}	2.02±0.07^{c}	2.35±0.10^{a}	1.98±0.05^{c}	2.44±0.11^{a}	2.41±0.01^{a}	0.060	0.010
Egg shape index	1.31±0.01	1.29±0.01	1.31±0.02	1.30±0.01	1.29±0.02	1.28±0.01	1.28±0.03	1.28±0.04	0.014	0.321
Eggshell strength (kg)	4.63±0.45	4.28±0.65	4.92±0.38	4.32±0.32	4.67±0.46	4.50±0.52	3.85±0.83	4.13±0.60	0.337	0.755
Yolk color	5.33±0.84	5.83±0.54	5.50±0.50	5.40±0.40	5.83±0.54	5.00±0.45	4.83±1.17	4.83±1.17	0.404	0.791
Haugh unit	85.83±1.37	81.56±1.87	85.36±1.31	85.46±2.47	82.28±2.32	87.14±1.87	84.82±7.28	82.90±4.87	1.948	0.563

[1] Values are mean ± standard error (SE) of 5 replicate pens per treatment for the 8-week experimental period.

[2] SEM=standard error of the mean

[a-b] Values with the same or not letter superscripts mean no significant difference(P>0.05), while with different letter superscripts mean significant difference(P<0.05).

Table S6. Fatty acids composition of experimental diets[1](mg/g, as-is basis).

Items	Control	Chia seed supplemental levels (%)						
		3	6	9	12	15	18	21
C16: 0	8.69±0.99	8.92±1.01	8.46±1.00	9.62±0.36	10.47±0.52	10.37±0.41	10.57±0.36	10.86±1.03
C18: 0	2.50±0.31	2.55±0.27	2.66±0.35	3.07±0.16	3.36±0.19	3.72±0.15	3.72±0.15	3.83±0.46
C18: 1	15.09±1.95	15.38±1.20	13.61±2.09	14.95±0.37	16.05±0.92	16.49±0.87	15.88±0.75	16.33±2.03
C18: 2 ω6	28.85±3.39	28.87±2.53	26.21±3.77	29.40±0.69	30.31±1.20	30.83±0.99	33.20±2.62	32.16±3.25
C18: 3 ω3	3.90±0.41	6.05±1.01	13.55±2.79	19.70±1.58	28.25±2.35	29.07±1.82	35.23±1.02	38.81±6.30

[1] Values are mean ± standard error (SE), n=3.

[a-b] Values with the same or not letter superscripts mean no significant difference(P>0.05), while with different letter superscripts mean significant difference(P<0.05).

Table S7. Effect of dietary chia seed levels on yolk, albumen, and eggshell content in eggs for laying hens after 8 weeks of feeding.[1]

Items	Control	Chia seed supplemental levels (%)							SEM[2]	*P*-value
		3	6	9	12	15	18	21		
Eggshell percentage (%)	13.47±0.44	12.78±0.44	13.06±0.30	12.63±0.19	13.11±0.21	12.85±0.51	11.70±0.26	12.76±0.24	0.517	0.053
Yolk percentage (%)	25.67±0.57	25.71±0.50	24.61±0.50	25.69±0.91	25.78±0.97	25.12±0.56	24.94±0.48	25.69±0.51	0.446	0.833
Albumen percentage (%)	60.85±0.68	61.52±0.86	62.34±0.49	61.68±0.87	61.11±1.01	62.03±0.96	63.36±0.53	61.56±0.70	0.783	0.426
Egg yolk ratio	0.42±0.01	0.42±0.01	0.40±0.01	0.42±0.02	0.42±0.02	0.41±0.01	0.39±0.01	0.42±0.01	0.012	0.724

[1] Values are mean ± standard error (SE) of 5 replicate pens per treatment for the 8-week experimental period.

[2] SEM=standard error of the mean

[a-b] Values with the same or not letter superscripts mean no significant difference(P>0.05), while with different letter superscripts mean significant difference(P<0.05).
